# Measles – retrospective study of cases reported in a national notification system, Czech Republic, 2018–2024

**DOI:** 10.1186/s12889-026-27042-8

**Published:** 2026-03-25

**Authors:** Monika Liptáková, Marek Malý, Michaela Špačková, Radomíra Limberková, Lada Svobodová, Jan Kynčl

**Affiliations:** 1 Department of Infectious Diseases Epidemiology, Centre for Epidemiology and Microbiology, National Institute of Public Health, Prague, Czech Republic; 2 Department of Biostatistics, National Institute of Public Health, Prague, Czech Republic; 3 National Reference Laboratory for Measles, Mumps, Rubella and Parvovirus B19, Centre for Epidemiology and Microbiology, National Institute of Public Health, Prague, Czech Republic; 4 Department of Epidemiology and Biostatistics, Third Faculty of Medicine, Charles University, Prague, Czech Republic

**Keywords:** Measles, Measles complications, Vaccination, Vaccination coverage

## Abstract

**Background:**

In response to the recent increase in measles cases and outbreaks across Europe, Czech surveillance data were analysed. The aim was also to evaluate the effect of vaccination on measles complications.

**Methods:**

Cases of measles reported to the national surveillance system according to the EU case definition (2018) between 2018–2024, were analysed. Demographic characteristics were assessed using descriptive analysis (counts and proportions). Logistic regression with an odds ratio (OR) supplemented with the 95% confidence interval (95% CI) was calculated to measure the association between vaccination status and the occurrence of complications.

**Results:**

A total of 791 measles cases were reported during the study period, of which 762 (96%) were laboratory confirmed, 29 (4%) were probable and 416 (53%) were male. Most cases (546; 69%) occurred in 2019. Half of the cases (*n* = 395, 50%) were outbreak related. The median age of cases was 33 years (range: 0–54). In total, 109 (14%) measles cases were imported (62 from Ukraine and smaller numbers from 25 other countries; 57 of the imported cases were unvaccinated) and 523 cases (70%) were hospitalised. In total, 330 (42%) of all cases were known to have been vaccinated, of which 205 (62%) with two or three doses and 75 (23%) with only one dose. Of 88 complications (11% of all cases) was pneumonia most frequent (n = 19; 2%). The highest proportion of complications occurred in infants (< 1 year [too young to be vaccinated]; 28%) and in children aged 1–4 years (20%). Compared to unvaccinated cases, two and more vaccine doses significantly reduced odds of any complications (OR = 0.44, 95% CI: 0.24–0.81, *p* = 0.008).

**Conclusions:**

Within analysed 7-year period most cases were detected pre-pandemic in 2018 and 2019. It is likely that restrictive measures limited further epidemic potential. Isolation of measles case is mandatory in Czech Republic; therefore, this criterion does not reflect the clinical severity of cases. Having this limitation in mind, the effect of vaccination against measles complications only was demonstrated. To prevent further cases, increased awareness and promotion of measles vaccination is recommended for all individuals, including travellers, as a substantial proportion of cases were unvaccinated.

**Supplementary Information:**

The online version contains supplementary material available at 10.1186/s12889-026-27042-8.

## Background

Measles is a severe highly infectious exanthematous viral disease which attributes significantly to childhood mortality although vaccination is available globally for decades. The disease is under the elimination programme of the World Health Organization (WHO) [1]. However, measles epidemiological situation has been unfavourable recently, globally and in Europe as well. Causes of such evolvement are combination of decreasing vaccination coverage (VC), importation from still endemic countries and the waning immunity [2, 3].

In 2024, the WHO European Region reported 127,350 measles cases, doubling the number recorded in 2023 and representing the highest incidence since 1997 [4]. This resurgence followed years of overall decline; after reaching a historic low of 4,440 cases in 2016, measles incidence increased in 2018–2019 and rose sharply following disruptions to surveillance system during the COVID-19 pandemic [4]. Children under five accounted for more than 40% of cases, and over half of patients required hospitalisation, indicating a substantial public health burden. The highest number of cases for 2024 was reported in Romania (30,692) and Kazakhstan (more than 28,000). This resurgence has been largely attributed to declining vaccination coverage and persistent immunity gaps that facilitate ongoing transmission [4]. Similar patterns in measles notifications have been reported by the European Centre for Disease Prevention and Control (ECDC): in 2024, the European Union (EU)/the European Economic Area (EEA) countries recorded 32,212 cases, with children under five being the most affected age group and a high proportion of unvaccinated individuals [5].

In the context of the recent measles resurgence across Europe, routine childhood immunization remains the cornerstone of prevention, though schedules and coverage vary between countries [6, 7]. Although WHO recommends two doses of a measles-containing vaccine with at least 95% coverage to achieve herd immunity, most EU/EEA countries have struggled to reach this threshold in recent years, resulting in the accumulation of susceptible individuals. In 2023, only a minority of countries reached ≥ 95% coverage with two doses, and several reported decline in both doses compared with pre-pandemic levels, creating immunity gaps that allow outbreaks to occur [6, 7]. Moreover, in 2023, approximately 500,000 children missed their first dose due to access and acceptance challenges [8]. Consequently, recent measles outbreaks underscore the need for supplementary and catch-up immunization strategies to prevent ongoing transmission and protect vulnerable populations [5, 9].

Czech Republic (CZ) represents a setting with a historically strong measles vaccination programme yet facing recent decline in coverage as well. In former Czechoslovakia, measles vaccination was introduced in 1969 as a single-dose schedule, followed by the implementation of a two-dose programme in 1982, and the trivalent measles-mumps-rubella (MMR) vaccine was introduced in 1995. Vaccination coverage remained above 95% for many years; however, since 2014 a gradual decline has been observed. An administrative survey conducted in 2017 revealed a nationwide coverage of only 83.5% among three-year-old children vaccinated with two doses of MMR, indicating the emergence of immunity gaps as well [2, 10]. From January 2018, schedule was changed to administer first dose of MMR vaccine at 13–18 months of age and second dose at 5–6 years of age [11].

In response to the recent increase in measles cases and reported outbreaks across Europe, Czech surveillance data were analysed. The aim of the study was also to assess the effect of vaccination on measles complications based on measles cases reported in 2018–2024.

## Methods

### Czech notification and surveillance system

Notification of measles into the national electronic surveillance system (ISIN) is mandatory in CZ [12]. The surveillance system is nationwide and case based. All physicians and laboratories who first diagnose the patient must report case data to the Regional Public Health Authority (RPHA) who further performs epidemiological investigation and enter the information into ISIN. The National Institute of Public Health collaborates on data validation and analyses and reports data internationally.

### Study population

All data of measles cases notified in ISIN under code “B05” of the 10th Revision of International Classification of Diseases (ICD-10 version) from 1 st January 2018 to 31 st December 2024 were retrieved from the ISIN and anonymised. Validity of data was processed by checking for duplicities and data integrity. A measles case reporting definitions are harmonised with the Commission Implementing Decision (EU) 2018/945 requirements in CZ; as confirmed case any person not recently vaccinated and meeting the clinical and the laboratory criteria and as probable case as any person meeting the clinical criteria with an epidemiological link. Possible cases and cases aged 55 years and older (cohort ineligible for vaccination) were excluded from the measles analysis.

Laboratory testing was performed in local medical laboratories and further specification, or genotyping was performed in the National Reference Laboratory for Measles, Mumps, Rubella and Parvovirus B19 (NRL).

A case was defined as vaccinated if the patient received at least one dose of measles vaccine more than 14 days before the symptom’s onset, which cutoff we decided based on the mean incubation period and expectation of at least partial onset of immunity after vaccination. The case was defined as partially vaccinated if having had only one dose of MMR vaccine or fully vaccinated receiving two and more doses of an appropriate vaccine. The vaccination status was obtained from paper or electronic vaccination records (both when available), or from medical documentation, or, if these were unavailable, from the patient’s own medical history (self-reported anamnesis).

### Data analysis

A detailed descriptive analysis of available data of measles cases was performed. Following variables were included: age, sex, year of reporting, administrative region, vaccination status, number of doses received by case, date of vaccination, complications, hospitalisation, clinical symptoms, outcome (alive/deceased), EU case classification (confirmed, probable), type of case (outbreak/sporadic, imported/autochthonous), country of importation, laboratory methods used (polymerase chain reaction [PCR] or serology IgM and/or IgG testing, genotyping if performed), type of sample and laboratory results. Complication types were defined using ICD-10 coding at the four-digit level (e.g., B05.0–B05.8). Notification rates were calculated based on mid-year population data published by the Czech Statistical Office [13].

Epidemiological characteristics of the cases were analysed using absolute and relative frequencies. Fisher’s exact test was used to examine the significance of the association between the two characteristics.

Timeliness of reporting was analysed as the interval between symptom onset and 1) the date of first specimen collection (T1 = diagnostic delay), and 2) the date of the RPHA receiving notification (T2 = notification delay). T1 could serve as an estimate for the first visit to a healthcare facility. It is a signal as to whether measles was also considered as part of the differential diagnosis and T2 also for implementation of early response measures.

To assess the effect of vaccination on the occurrence of measles complications in cases that were fully vaccinated and partially vaccinated compared to unvaccinated the univariate logistic regression model was used. Cases with unknown vaccination status and vaccinated cases with an unknown number of vaccine doses were excluded.

Three different outcomes were further considered: any complications (any reported measles complications), solely measles-related pneumonia (B05.2) and solely gastrointestinal measles complications (B05.4). Results were displayed as unadjusted odds ratios (OR) with 95% confidence intervals (95% CI). In the case of separation problems related to small numbers, logistic regression model was fitted using the Firth's bias reduction method [14].

Czech legislation mandates hospital isolation for all measles cases, regardless of disease severity, therefore we did not consider hospitalisation as an outcome in analytical part of the study.

Findings with *p*-values less than 0.05 were considered statistically significant. Analysis was performed in STATA, version 17 (StataCorp LLC, College Station, Texas, USA).

A sensitivity analysis was conducted to examine the results of the analysis under alternative imputation scenarios for missing vaccination status data and to assess the robustness of the findings against potential bias. Three scenarios were used:

1. All cases with missing vaccination status data were considered to have been vaccinated with two doses,
2. All cases with missing vaccination status data were considered unvaccinated,
3. Missing vaccination status data were imputed randomly, but so that the percentage representation of each option, i.e. 1 or 2 doses for individuals who were vaccinated but with an unknown number of doses, and 0, 1, or 2 doses for individuals with completely unknown vaccination status, corresponded to the representation in patients with available information. Ten sets of randomly imputed data were prepared.

## Results

A total of 762 (96.3%) were confirmed and 29 (3.7%) were probable, i.e. epidemiologically linked to a confirmed case. Clinical symptoms of cases, as reported, included: exanthema in 75.4% (585/776), cough in 56.3% (437/776), fever > 38 °C in 55.7% (395/709), running nose in 39.2% (304/776), conjunctivitis in 25.6% (199/776) and Koplik spots in 14.4% (112/776).

During the study period, a total of 791 measles cases were reported in the CZ, distributed unevenly over time Supplement 1. Most cases were notified in 2019 (n = 546; 69.0% of all cases, 7.5/100,000 inhabitants) and 2018 (*n* = 205, 25.9%, 2.8/100,000 inhabitants), followed by a significant decline in subsequent years to 0 cases reported in 2021 and 2022 and small increase in 2024 (*n* = 35, 4.4%, 0.5/100,000 inhabitants), Supplement 1. The mean age of cases at the disease onset was 29 years, median 33 years (range 0–54). The youngest age group (infants and 1–4 years) was the most affected. Cases were reported from all Czech regions, with the highest notification rate observed in Prague, Pardubice and Hradec Králové regions, Supplement 1.

### Vaccination status

Of all cases, 330 (41.7%) reported to have been vaccinated. Of vaccinated, 202 received two and 3 cases received 3 doses of vaccine, therefore 205 (62.1%) cases were considered fully vaccinated. Another 316 (40.0%) were unvaccinated and 145 (18.3%) had unknown vaccination status, Table 1.

**Table 1:** Vaccination status of measles cases by age groups, Czech Republic, 2018–2024

Age	Vaccination status						Total
	**Vaccinated**				**Unvaccinated**	**Unknown**	
	**Three doses**	**Two doses**	**One dose**	**Unknown number of doses**			
0	0	0	0	0	33	7	40
1–4	0	0	4	2	65	18	89
5–9	0	3	1	1	14	3	22
10–14	1	11	1	0	7	5	25
15–19	0	18	2	4	12	7	43
20–24	1	29	5	0	26	7	68
25–34	1	54	2	6	42	30	135
35–44	0	67	35	24	60	38	224
45–54	0	20	25	13	57	30	145
**Total**	**3**	**202**	**75**	**50**	**316**	**145**	**791**

Data source: Czech infectious diseases notification system (ISIN)

Eighty-nine (88.9) percent of the cases had no clinical complications. Among complicated cases (*n* = 88), the most frequently reported complication was pneumonia (*n* = 19, 2.4%), followed by gastrointestinal complications (*n* = 18, 2.3%), otitis (*n* = 6, 0.8%), and other unspecified complications (*n* = 45, 5.7%), Table 2. Hospitalisation status was known in 753 cases and of these, 523 (69.5%) were hospitalised (Tables 2 and 3). The proportion of hospitalised patients was slightly higher (*p* = 0.057) in males (290/400, i.e. 72.5%) than in females (233/353, i.e. 66.01%). The highest proportion of complications occurred in infants (< 1 year [too young to be vaccinated]; n = 11, 27.5%) and children aged 1–4 years (n = 18, 20.2%). Males accounted for 52.3% among complicated cases (46/88) and 55.5% of hospitalisations (290/523). The highest proportion of measles hospitalisations was reported in the age group 0 and 1–4 years (83.8% and 83.7% respectively) (Table 3).

**Table 2:** Measles complications, hospitalisation and vaccination, Czech Republic, 2018–2024

Characteristic	Vaccination status						Total
	**Vaccinated**				**Unvaccinated**	**Unknown**	
	**Three doses**	**Two doses**	**One dose**	**Unknown number of doses**			
Total number of cases	3	202	75	50	316	145	791
Complications							
None	2	188	70	44	268	131	703
Pneumonia	0	2	0	3	10	4	19
Gastrointestinal	0	4	1	1	6	6	18
Otitis	0	0	0	0	5	1	6
Other complications	1	8	4	2	27	3	45
Hospitalisation							
Yes	2	118	51	27	231	94	523
No	1	76	18	21	70	44	230
Unknown	0	8	6	2	15	7	38

Data source: Czech infectious diseases notification system (ISIN)

**Table 3:** Proportion of measles complications and hospitalisation by age group, Czech Republic, 2018–2024

Age group (years)	Complications			Hospitalisation		
	number of cases with complication	number of measles cases	Proportion (%)	number of hospitalised cases	number of measles cases	Proportion (%)
0	11	40	27.50	31	37	83.78
1–4	18	89	20.22	72	86	83.72
5–9	1	22	4.55	11	22	50.00
10–14	5	25	20.00	17	24	70.83
15–19	3	43	6.98	33	41	80.49
20–24	5	68	7.35	52	64	81.25
25–34	13	135	9.63	93	127	73.23
35–44	16	224	7.14	129	211	61.14
45–54	16	145	11.03	85	141	60.28
Total	**88**	**791**	**11.13**	**523**	**753**	**69.46**

Data source: Czech infectious diseases notification system (ISIN)

During the study period 2018–2024, number of measles cases notified by the year of reporting was: 205, 546, 4, 0, 0, 1 and 35 (Supplement 1). Three cases with a year of symptoms onset in 2017 were reported in 2018. The most measles cases by the year of onset were reported in 2019 and 2018 (Table 4).

**Table 4:** Measles complications and hospitalisation by year of onset, Czech Republic, 2018–2024

Year of symptoms onset	2017	2018	2019	2020	2021	2022	2023	2024
Complications (*n* = 88)	0	21	64	0	0	0	0	3
Hospitalisation (*n* = 523)	2	147	354	2	0	0	1	17
Number of measles cases (*n* = 791)	3	212	536	4	0	0	1	35

Data source: Czech infectious diseases notification system (ISIN)

### Association between vaccination and complications

Among measles cases with complications, 22.1% of individuals received two and more doses of MMR vaccine, while among cases without complications, 36.0% received two and more doses of the vaccine (*p* = 0.010) (Table 5).

**Table 5:** Proportion of measles cases with complications, Czech Republic, 2018–2024

Any complications	Vaccination status			Total
	**unvaccinated**	**partially vaccinated**	**fully vaccinated**	
no	268	70	190	528
proportion (%)	50.76	13.26	35.98	100.00
yes	48	5	15	68
proportion (%)	70.59	7.35	22.06	100.00
Total	316	75	205	596
proportion (%)	53.02	12.58	34.40	100.00

Data source: Czech infectious diseases notification system (ISIN)

partially vaccinated = one dose of MMR vaccine, fully vaccinated = two and more doses

Two and more doses of vaccine significantly (p = 0.008) reduced the risk of any complications compared to unvaccinated cases, resulting in an OR of 0.44 (95% CI: 0.24, 0.81) (Table 6).

**Table 6:** Number of measles cases by complications and vaccination status, Czech Republic, 2018–2024

Type of complications	Vaccination by number of doses	Number of cases	Number of cases with complications (%)	Odds ratio (95% CI)	p value
Any complication	unvaccinated	316	48 (15.2)	Ref	
	1	75	5 (6.7)	0.40 (0.15, 1.04)	0.060
	≥ 2	205	15 (7.3)	0.44 (0.24, 0.81)	0.008
Pneumonia	unvaccinated	316	10 (3.2)	Ref	
	1	75	0 (0.0)	0.19 (0.01, 3.34)	0.258
	≥ 2	205	2 (1.0)	0.36 (0.09, 1.44)	0.148
Gastrointestinal	unvaccinated	316	6 (1.9)	Ref	
	1	75	1 (1.3)	0.96 (0.16, 5.78)	0.966
	≥ 2	205	4 (2.0)	1.07 (0.32, 3.60)	0.917

Data source: Czech infectious diseases notification system (ISIN)

Ref – reference category (unvaccinated)

A sensitivity analysis of the results in Table 6 was performed to assess bias, using the same inclusion and exclusion criteria. When all cases with missing vaccination status data were considered to have been vaccinated with two doses (scenario 1), two and more doses of vaccine significantly (*p* = 0.008) reduced the risk of any complications compared to unvaccinated cases, resulting in an OR of 0.54 (95% CI: 0.34, 0.85). When all cases with missing vaccination status data were assumed unvaccinated (scenario 2), two and more doses of vaccine significantly (*p* = 0.026) reduced the risk of any complications compared to unvaccinated cases, resulting in an OR of 0.51 (95% CI: 0.29, 0.92). The results of ten variants of logistic regression calculation after random imputation of missing vaccination status data (scenario 3) provided OR values ranging from 0.49 to 0.59 and p-values from 0.005 to 0.040. In all three scenarios, the effect of two doses was always statistically significant, but the ORs were worse than from the incomplete data.

### Import and outbreak-related data

A total of 109 (13.8%) measles cases were imported, most of them from Ukraine (62, 56.9%), followed by Austria (4, 3.7%), Croatia (4, 3.7%), Vietnam (4, 3.7%) and smaller numbers from 22 other countries. In total, 57 (52.3%) imported cases were unvaccinated.

A total of 395 (49.9%) outbreak-related cases were reported in the ISIN, originating from six outbreaks (Table 7).

**Table 7:** Summary of measles outbreak-related data, Czech Republic, 2018–2024

Region	Year	Number of cases	Index case origin	Age range (mean)	Vaccination status			Settings
					vaccinated	unvaccinated	unknown	
Prague	2018–2019	243	Ukraine	0–54 (28)	84	84	75	community
Hradec Králové	2019	33	Vietnam	22–54 (38)	23	6	4	workplace
South Moravian	2019	7	Switzerland	30–49 (39)	4	3	0	workplace
Moravian-Silesian	2019	9	unknown	21–54 (38)	6	3	0	hospital
Moravian-Silesian	2019	91	unknown	0–54 (25)	38	53	0	community
South Bohemian	2024	12	Germany	3–54 (27)	0	6	6	school

Data source: Czech infectious diseases notification system (ISIN)

The highest proportion of measles cases in outbreaks was reported in the age group 35–44 years (114, 29%), followed by the age group 45–54 (69, 17%), and 25–34 years (63, 16%).

Information from the hospital settings outbreak report:

Out of nine measles cases, were infected six employees of the hospital, two students of medical faculty and a one family member (of the index case). During the epidemiological investigation, in total 173 people were exposed and tested for measles (168 people for IgM serology, four of them were positive and reported as measles cases; and five people were tested by PCR and five were positive and reported as measles cases). Nine cases were laboratory confirmed. Twenty susceptible people were quarantined, five were immediately vaccinated. Susceptible employees were vaccinated at the employer's expense after the quarantine. Out of nine measles cases, four people were hospitalised in department of infectious diseases and five were isolated at home. The source of the disease (index case) was a nurse whose measles illness was misdiagnosed as a scarlet fever; therefore, appropriate response measures could not be taken immediately.

Information from the workplace settings outbreak report:

Index case was an employee travelling abroad (Vietnam), who had health problems, but the disease was not detected in time and was only proven subsequently, in connection with measles outbreak among other employees. The index case was initially misdiagnosed as suspected dengue fever, then later confirmed as measles. Of the total number of 1027 exposed (751 employees at workplace and 276 of family members and other people at risk), 33 measles cases were notified. First case occurred in administrative part of workplace, but then measles spread into manufacturing part. In total, 33 cases were hospitalised (average length of hospitalisation was 7 days). Based on the EU case definition: 30 cases were confirmed, three probable.

Information from the school settings outbreak report [15]:

Index case was an unvaccinated girl who probably became infected in Germany, and the infection was introduced into the school facility, where there are also unvaccinated children, and the infection gradually spread to adult susceptible contacts. Of the total number of 12 cases, six were children (50%), all unvaccinated. Ten cases were laboratory confirmed (eight cases by PCR, two by IgM serology in the NRL) and two cases were probable. Four cases were hospitalised (average length of hospitalisation was 5 days), eight were isolated at home. In total, 127 susceptible individuals were identified, and their immune status was examined by general practitioners. Post-exposure prophylaxis was administered in eight individuals, and one unvaccinated infant was given immunoglobulin. Lesson learned: when investigating suspected measles cases, blood sampling for antibody testing is not sufficient; for early diagnosis, PCR testing of nasopharyngeal swabs is necessary, which must be provided in the field [15].

## Laboratory data

In our study, serology (449/772, i.e. 58.2% of samples) as a laboratory method prevailed over PCR testing (315/772, i.e. 40.8% of samples). Most tested laboratory specimen were serum in 496/772 cases (64.2%) and nasopharyngeal swab in 265/772 cases (34.3%, Table 8). A total of 393/777 cases (50.6%) were confirmed in the NRL, 384/777 cases (49.4%) were confirmed in regional laboratories from all regions in the CZ and in 14 cases the information was missing. The results of serologic tests (IgM and/or IgG) were positive in 709/737 cases (96%), borderline in 2 cases (< 1%), negative in 16 cases (2%), inconclusive in 10 cases (1%), and missing in 54 cases. However, all these cases were reported in the ISIN. The most missing data were on immunoglobulin IgM (346 cases) or IgG (372 cases) serology (44% and 47%, respectively). Information about genotypes was obtained from testing performed at the NRL and their registry. During the study period, following genotypes were confirmed in the NRL: in 2018: B3 (5 cases), D8 (35 cases); in 2019: B3 (10 cases), D8 (187 cases); in 2023: D8 (1 case), in 2024: B3 (4 cases) and D8 in 6 cases.

**Table 8:** Measles data on laboratory methods and specimens, Czech Republic, 2018–2024

**Method**	**2018**	**2019**	**2020**	**2023**	**2024**	**Total**
serology	158	271	2	1	17	449
PCR	37	260	2	0	16	315
unknown	3	5	0	0	0	8
missing	7	10	0	0	2	19
Total	205	546	4	1	35	791

**Material**	**2018**	**2019**	**2020**	**2023**	**2024**	**Total**
serum	166	308	2	1	19	496
swab	32	218	2	0	13	265
urine	0	10	0	0	0	10
unknown	1	0	0	0	0	1
missing	6	10	0	0	3	19
Total	205	546	4	1	35	791

Data source: Czech infectious diseases notification system (ISIN)

### Timeliness

Timeliness data were analysed, the median interval for diagnostic delay (T1, 777/791) was five days (range: 0–74 days; lower and upper quartiles [Q1, Q3]: 3, 6). The median interval for notification delay (T2, in all 791 cases) was eight days (range: 0–90; [Q1, Q3]: 4, 8).

## Discussion

In our study, the measles notification rate was the highest among infants and young children (< 5 years), as was demonstrated by many studies already [5, 16–18]. Similarly, the highest proportion of measles complications was reported in infants (28%) [18] and in children aged 1–4 years (20%).

In CZ, measles has spread mainly among young adults during the last outbreaks, similarly to Italy where 75% of reported measles cases were ≥ 15 years of age [17]. During the COVID-19 pandemic, the measles notification rate in CZ decreased to zero, an alike phenomenon was also observed in most EU/EEA countries. Subsequently, the number of cases began to rise, following a pattern similar to that seen in other countries [5].

The data showed that complications were less frequent than in Italy (35%) and the United States (37%) [17, 19], suggesting that Czech surveillance system also captured milder cases. The most frequent complication was pneumonia, similarly, as documented in other high-income countries, where also otitis and diarrhoea were reported [5, 18, 20].

The proportion of hospitalisation for measles cases varies substantially among high-income countries, e.g. 7% in Israel (4,158 notified cases), 18–20% in the USA (4,056 confirmed cases), 43% in Belgium and 43% in Italy (over 4,400 reported cases) [17, 19, 21–23]. The EU/EEA data in 2024 showed that 79% of reported measles cases required hospitalisation, 80% in 2023, 33% in 2022 and 55% in 2019 [5]. Based on the national legislation (Annex No. 2 to Decree No. 306/2012 Coll., List of infectious diseases for which isolation is ordered in inpatient wards of hospitals or medical institutions, and diseases for which treatment is mandatory), measles case should be isolated in the hospital. Patient isolation lasts for four days after the appearance of a rash [24] to reduce the exposure of other contacts at high risk. In our study, 71% of unvaccinated participants with measles had complications, while among one- or two-dose vaccine recipients with measles the complication rate was about 7–22%. The reason for that difference is in the vaccination status itself, as observed in a Spanish study, where 'severity and clinical presentation were milder among the vaccinated, though some cases still required hospitalisation due to complications' [25]. It could be more plausible that clinicians are less strict about isolating patients who have received two vaccine doses, which could explain the lower hospitalisation rate in vaccinated cases. Disease severity can be estimated based on measles complications reported in ISIN.

We did not focus on comparison of clinical symptoms and the laboratory results between vaccinated and unvaccinated patient groups. This topic was recently studied in 230 patients with measles who were hospitalised or examined at Bulovka University Hospital in Prague between January 2018 and December 2019 [26]. This number represents more than a quarter (29%) of all cases in CZ reported to ISIN in 2018 and 2019. Only two thirds of the fully vaccinated patients from Bulovka Hospital developed typical measles exanthema and almost 30% of patients vaccinated with one or two doses did not meet the clinical case criteria. Therefore, less strict clinical criteria should be considered in highly vaccinated populations in order to detect as many measles cases as possible [26].

Data from studies nonetheless stress that over long term two doses of the MMR vaccine are highly effective, while also waning immunity should be considered in the measles elimination process [27, 28].

Prompt reporting (≤ 24 h) of suspected cases and immunization of all susceptible contacts is highlighted to limit spread [29], but based on timeliness results in CZ, this was not always feasible, several outbreaks and spreading into the community were reported.

The last seroepidemiological study conducted in 2013 in CZ revealed a 23% susceptibility to measles infection among persons vaccinated in the early period of vaccination [2, 30]. To set up targeted and effective response measures, it is necessary to monitor the immune status of the population, ideally at five-year intervals. The serological surveys would provide valuable information on the persistence of post-vaccination antibodies after childhood vaccination, not only for measles; data are useful for evaluation of vaccination strategies as well. It is also necessary to ensure a detailed epidemiological history and, in the case of an exanthematous disease, also consider measles in the differential diagnosis, taking into account that measles exanthema in previously vaccinated persons, can have a milder clinical course, as "vaccine-modified measles" [2, 26, 31, 32].

Based on the internal RPHA outbreak report from the workplace, most people of working age did not have sufficient levels of antibodies against measles. Possible changes in the vaccination calendar related to this remain a question for the future. In this context, the question arises whether, it would not be appropriate to revaccinate against measles [33–35] (preferably with the guarantee of a state subsidy), especially people who work in jobs that ensure the basic functioning of the state, such as not only healthcare workers.

International travellers (imported cases) accounted for a small proportion of measles cases, as published [5, 36], which is consistent with our results. Unvaccinated individuals travelling abroad, unvaccinated health care workers and other risk groups can transmit measles to susceptible individuals; targeted revaccination of these risk groups is expected to help prevent the spread of measles during outbreaks [37].

The relatively large outbreaks among young adults in CZ might have been caused by waning immunity. Such assumption can be supported by serosurveys performed in CZ [38–41] and in abroad [42]; they indicated a significant decline in immunity in older vaccinated cohorts [38–41]. Primary vaccine failure may also be a reason for measles despite earlier vaccination. Based on available data, measles cases in our cohort were not vaccinated with the Leningrad-16-vaccine.

The childhood immunisation programme against MMR in CZ has changed since January 2018, and not enough data for the evaluation of the new schedule was available. As of 31 December 2020, more than 95% of those born in 2017 received at least one dose of the MMR vaccine in different regions.

Since vaccination is the only preventive measure, as no specific treatment exists for measles, it is essential to emphasize the importance of vaccination in preventing disease severity, especially in unvaccinated or vaccine hesitant population. Strengthening vaccination coverage with two doses of the MMR vaccine is crucial, especially in regions with lower uptake. However, the administrative survey of vaccination in CZ was discontinued as of 1 January 2022, and its results have since been replaced by vaccination coverage estimates based on data from health insurance companies [2, 10, 43].

We cannot rule out that some cases were detected and reported too late for postexposure vaccination due to timeliness results in CZ. Nevertheless, information about postexposure prophylaxis was not the subject of our study.

People born before 1969, before the introduction of nationwide measles vaccination, are considered immune, similar results were observed in neighbouring countries [42, 44]. Information about the vaccine administered in the past can be demonstrated based on laboratory testing of IgG antibody avidity [45].

Proper and early epidemiological investigation is crucial for identification of transmission patterns to improve contact tracing and prevent the spread of the disease. We recommend enhancing doctors’ awareness and improving laboratory confirmation procedures to achieve early detection of measles cases for better outbreak control. As part of ongoing surveillance, it is necessary to maintain high data quality when entering it in the notification system.

The main limitation of the study is the incompleteness of reported vaccination data, with many cases lacking information on vaccination status and the number of administered doses. As the national electronic vaccination register has been launched in CZ in 2023, data on coverage with the second MMR dose remain limited [43]. Of those with unknown vaccination status, some were likely vaccinated, meaning that the 42% vaccination rate in measles cases observed across the study period may underestimate the true figure. Misclassification of vaccination status cannot be excluded due to recall bias, as parental recall tends to overestimate coverage, whereas written records often underestimate it. In addition, information on hospitalisation was typically recorded at the time of testing and may also have been underreported. Due to the national legislation, we cannot distinguish between hospitalisations for mandatory isolation and those due to disease severity, therefore we used in logistic regression only outcome “complications”.

Without renewed efforts to strengthen routine immunization, address vaccine acceptance issues, and close coverage gaps, the current heterogeneity in European vaccination programmes will continue to shape the epidemiological dynamics of measles, with ongoing outbreaks likely where population immunity remains insufficient [46].

## Conclusions

Our findings demonstrated that two doses of vaccine substantially and significantly reduced the risk of complications in measles cases compared with unvaccinated measles cases, confirming the protective effect of two-dose vaccination regimen against measles. As Czech legislation mandates hospital isolation for all measles cases, however, this criterion does not reflect the clinical severity of cases.

To monitor the effectiveness of vaccination programmes, a seroepidemiological study would be appropriate, as the last nationwide survey of measles antibodies was conducted in 2013. Further monitoring and research are also needed to evaluate the impact of the revised MMR vaccination schedule and to determine whether it provides greater impact. We recommend the continuation of routine childhood measles vaccination, the maintenance of high MMR coverage and efforts to increase coverage among individuals lacking immunity to measles in CZ. To prevent further cases, increased awareness and promotion of measles vaccination is recommended for all individuals, including travellers, as a substantial proportion of cases were unvaccinated.

## Supplementary Information

Additional file 1. Supplement 1. Distribution of measles cases aged 0-54 years by demographic characteristics, Czech Republic, 2018-2024.

## Data Availability

The datasets used and analysed during the current study are available from the corresponding author on reasonable request.
