# Supplementary material for: Measles – retrospective study of cases reported in a national notification system, Czech Republic, 2018–2024
Source: BMC Public Health. 2026 Mar 25;26:1451. doi: 10.1186/s12889-026-27042-8 (PMC13137613; doi:10.1186/s12889-026-27042-8)
Supplement: Supplementary file 1 — Additional file 1. Supplement 1. Distribution of measles cases aged 0-54 years by demographic characteristics, Czech Republic, 2018-2024. [file 12889_2026_27042_MOESM1_ESM.docx]

**Supplement 1.** Distribution of measles cases aged 0-54 years by demographic characteristics, Czech Republic, 2018-2024

| **Characteristic** | **Number of cases**  **(n=791)** | **Proportion (%)** | **Notification rate per 100,000 population** |
| --- | --- | --- | --- |
| **Year of reporting** | | | |
| 2018 | 205 | 25.9 | 2.8 |
| 2019 | 546 | 69.0 | 7.5 |
| 2020 | 4 | 0.5 | 0.1 |
| 2021 | 0 | 0 | 0.0 |
| 2022 | 0 | 0 | 0.0 |
| 2023 | 1 | 0.1 | <0.1 |
| 2024 | 35 | 4.4 | 0.5 |
| **Administrative region** | | | |
| Prague | 294 | 37.2 | 4.5 |
| Central Bohemian | 86 | 10.9 | 1.2 |
| South Bohemian | 37 | 4.7 | 1.2 |
| Plzeň | 46 | 5.8 | 1.6 |
| Karlovy Vary | 2 | 0.3 | 0.1 |
| Ústí nad Labem | 11 | 1.4 | 0.3 |
| Liberec | 13 | 1.6 | 0.6 |
| Hradec Králové | 59 | 7.5 | 2.3 |
| Pardubice | 66 | 8.3 | 2.7 |
| Vysočina | 11 | 1.4 | 0.5 |
| South Moravian | 36 | 4.6 | 0.6 |
| Olomouc | 5 | 0.6 | 0.2 |
| Zlín | 13 | 1.6 | 0.5 |
| Moravian-Silesian | 112 | 14.2 | 2.0 |
| **Gender** | | | |
| Male | 416 | 52.6 | 1.6 |
| Female | 375 | 47.4 | 1.5 |
| **Age group (years)** | | | |
| 0 | 40 | 5.1 | 5.4 |
| 1–4 | 89 | 11.3 | 2.8 |
| 5–9 | 22 | 2.8 | 0.5 |
| 10–14 | 25 | 3.2 | 0.6 |
| 15–19 | 43 | 5.4 | 1.2 |
| 20–24 | 68 | 8.6 | 2.0 |
| 25–34 | 135 | 17.1 | 1.5 |
| 35–44 | 224 | 28.3 | 2.0 |
| 45–54 | 145 | 18.3 | 1.3 |

*Data source:* Czech infectious diseases notification system (ISIN)
